# Structure-Function Correlation of the Human Central Retina

**DOI:** 10.1371/journal.pone.0012864

**Published:** 2010-09-22

**Authors:** Peter Charbel Issa, Eric Troeger, Robert Finger, Frank G. Holz, Robert Wilke, Hendrik P. N. Scholl

**Affiliations:** 1 Department of Ophthalmology, University of Bonn, Bonn, Germany; 2 Nuffield Laboratory of Ophthalmology, John Radcliffe Hospital, University of Oxford, Oxford, United Kingdom; 3 Biomedical Engineering Laboratory, Institute for Ophthalmic Research, Centre for Ophthalmology, University of Tuebingen, Tuebingen, Germany; 4 Graduate School of Biomedical Engineering, University of New South Wales, Sydney, Australia; 5 Wilmer Eye Institute, Johns Hopkins University, Baltimore, Maryland, United States of America; University of Oldenburg, Germany

## Abstract

**Background:**

The impact of retinal pathology detected by high-resolution imaging on vision remains largely unexplored. Therefore, the aim of the study was to achieve high-resolution structure-function correlation of the human macula in vivo.

**Methodology/Principal Findings:**

To obtain high-resolution tomographic and topographic images of the macula spectral-domain optical coherence tomography (SD-OCT) and confocal scanning laser ophthalmoscopy (cSLO), respectively, were used. Functional mapping of the macula was obtained by using fundus-controlled microperimetry. Custom software allowed for co-registration of the fundus mapped microperimetry coordinates with both SD-OCT and cSLO datasets. The method was applied in a cross-sectional observational study of retinal diseases and in a clinical trial investigating the effectiveness of intravitreal ranibizumab in macular telangietasia type 2. There was a significant relationship between outer retinal thickness and retinal sensitivity (p<0.001) and neurodegeneration leaving less than about 50 µm of parafoveal outer retinal thickness completely abolished light sensitivity. In contrast, functional preservation was found if neurodegeneration spared the photoreceptors, but caused quite extensive disruption of the inner retina. Longitudinal data revealed that small lesions affecting the photoreceptor layer typically precede functional detection but later cause severe loss of light sensitivity. Ranibizumab was shown to be ineffective to prevent such functional loss in macular telangietasia type 2.

**Conclusions/Significance:**

Since there is a general need for efficient monitoring of the effectiveness of therapy in neurodegenerative diseases of the retina and since SD-OCT imaging is becoming more widely available, surrogate endpoints derived from such structure-function correlation may become highly relevant in future clinical trials.

## Introduction

The human central retina, the macula, is both the anatomical structure responsible for fine detail vision and subject to disease causing irreversible blindness. Histology has long been the only means to investigate retinal morphology in health and disease, and thus, the functional impact of the pathologic changes of the retina largely remained unknown.

Recent developments in high-resolution *in vivo* imaging of the central retina such as spectral domain optical coherence tomography (SD-OCT) and confocal scanning laser ophthalmoscopy (cSLO) promise to enhance early diagnosis and objective evaluation of macular diseases. High-resolution OCT generates tomographic images similar to tissue sections and permits visualization of retinal morphology that had previously only been possible with histopathology [1], [2]. cSLO imaging provides detailed and high-contrast topographic images similar to photography and has become a powerful tool to monitor atrophic and dystrophic retinal diseases [3]. Investigating treatment effectiveness by high-resolution imaging has very recently gained tremendous importance because retinal pharmacotherapy, especially vascular endothelial growth factor A (VEGF-A) inhibition, has revolutionized therapy of macular diseases [4], [5], [6], [7], [8]. However, there remains a fundamental problem because the functional consequences of abnormalities detected with such high-resolution imaging technology are unclear. Therapeutic intervention in order to modify the retinal lesions detected with high-resolution imaging may only be justified if these abnormalities indeed cause functional loss.

Fundus-controlled microperimetry is a functional test allowing analysis of retinal sensitivity with a high spatial resolution [9]. It differs from standard perimetry techniques in that it is independent from eye movements during the examination. This is achieved by controlled projection of the test stimuli in constant topographic relation to retinal landmarks. An exact overlay of such functional mapping with high-resolution images of the retina would reveal the functional impact of microstructural alterations *in vivo*. Although simultaneous topographic (cSLO imaging) and tomographic (SD-OCT) retinal imaging in exact spatial correlation has recently been developed in one instrument [10], high-resolution structure-function correlation has not yet been achieved. Therefore, we aimed at a co-registration of quasi-histological and high-resolution functional mapping of the central retina. We studied the impact of neurodegenerative structural changes on visual function and investigated how this technique may add further insight into the outcome of pharmacological intervention.

The data show that retinal *in vivo* imaging with histology-like resolution and its exact co-registration with functional high-resolution mapping of the macula is now feasible. A method (MultiModalMapper) was developed that allows for accurate co-registration of the coordinate systems of fundus-controlled microperimetry, high-resolution 3D-OCT datasets and cSLO images. By translating the method into a clinical setting, the proof of concept is provided for the ability of the technique to generate unprecedented information on the functional impact of retinal pathology. The technique may not only increase the reliability of the interpretation of findings revealed by different modes of retinal imaging. It will also allow the use of high-resolution imaging techniques for estimating functional consequences of morphological alterations and assess treatment benefits in a more objective way.

## Results

### Accuracy of Co-registration of Functional Mapping and Retinal Imaging

**Figure 1:**
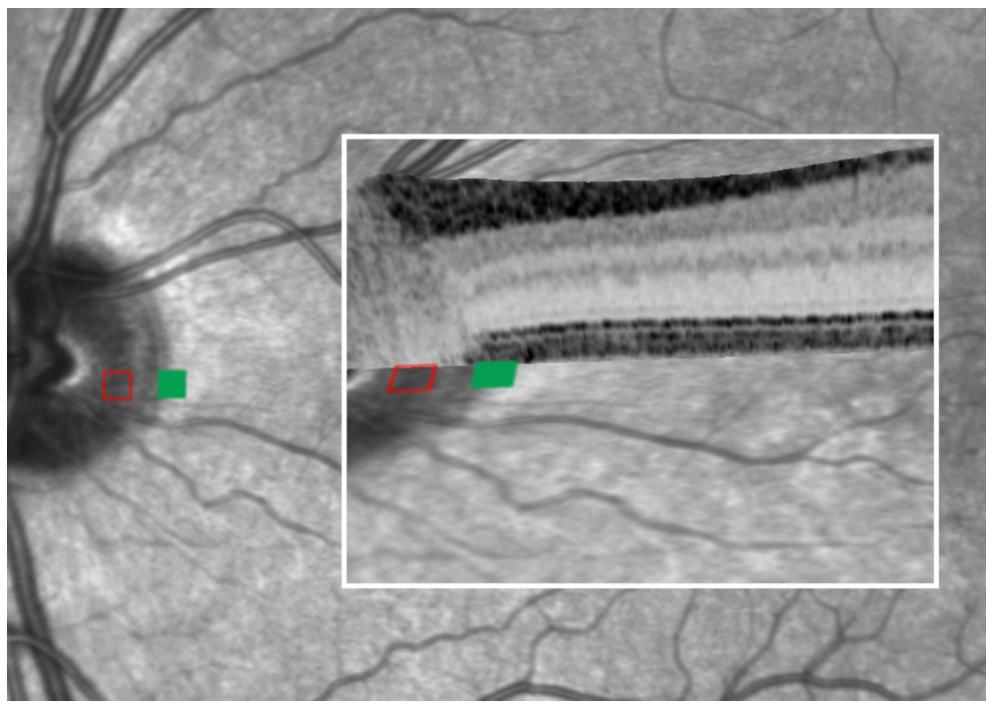
Ability of co-registration using the MultiModalMapper software to exactly map functional testing on anatomical recordings in a normal subject. The optic nerve head is the anatomical correlate for the physiological blind spot in the visual field. The hollow red square represents the brightest stimulus using microperimetry and was not detected by the subject (absolute scotoma) when projected onto the optic nerve head. In contrast, a much dimmer testing point (green square, 18dB decreased light intensity compared to the brightest stimulus) projected just outside the optic nerve area head was detected by the subject and shows normal light sensitivity. An overlay with the SD-OCT scan (inset, framed in white) shows the normal retinal layers within the area bordering the optic nerve head.

**Figure 2:**
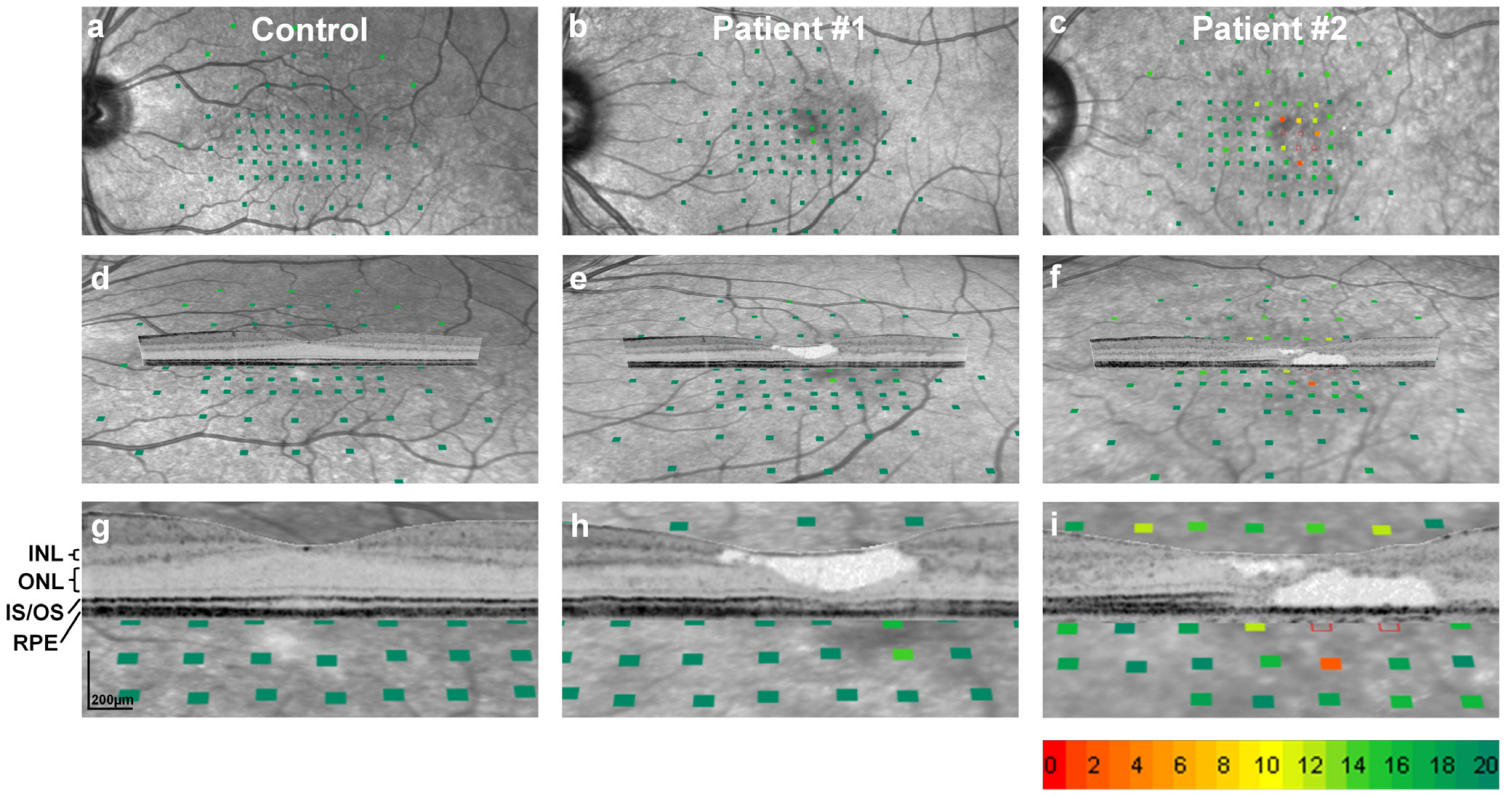
Correlation of topographic and tomographic retinal imaging with functional mapping. Threshold values of retinal sensitivity derived from microperimetry are presented according to the scale below panel i) in 2 dB steps. Normal sensitivity is indicated by green colour, and decreased sensitivity is indicated by red colour; open rectangles demonstrate absolute defects (as defined in Ref [11]). The first column illustrates findings in a normal subject, the middle and right column in two patients with macular telangiectasia type 2. The first row shows functional maps superimposed on near-infrared confocal scanning laser ophthalmoscopy (cSLO) images of the central retina. The second and third row (d–i) show the functional maps superimposed on the back-tilted cSLO images and a high-resolution spectral-domain optical coherence tomography (SD-OCT) scan. The lower panels (g-i) show enlarged cut-outs of the middle row (d–f). For the normal subject, selected anatomical layers of the retina are indicated for orientation on the left-hand side of panel g (INL: inner nuclear layer; ONL: outer nuclear layer; IS/OS: junction of the inner and outer photoreceptor segment; RPE: retinal pigment epithelium). The tissue defect within the inner retina (e,h) is associated with a normal retinal light sensitivity (green squares), whereas damage to the outer retina (the photoreceptor layers) was accompanied by a strong loss of retinal sensitivity (red filled and red open squares; f,i).

The quality of co-registration of retinal sensitivity maps and imaging (both tomographic and topographic) data is exemplified for the optic disc in a normal subject (Fig. 1). The optic disc area represents the physiologic blind spot which can be mapped using microperimetry as previously shown [9]. There was accurate co-registration showing that within the blind spot, there was no light sensitivity detectable, whereas just outside the optic disc, normal retinal sensitivity was measured. This correlated both topographically with the retinal area outside the optic disc on infrared reflectance image and with a normal outer retinal morphology as imaged by SD-OCT (Fig. 1, inlay). Structure-function correlation of the macula in a normal subject is shown in Fig. 2, (left column) and in the Video S1.

### Structure-Function Correlation in Macular Telangiectasia Type 2: Cross-sectional Data

In macular disease, well-defined features of structure-function correlation are exemplified in macular telangiectasia type 2. This neurodegenerative disease shares many similarities with age-related macular degeneration and diabetic retinopathy, together by far the most common causes of blindness in the western world. Well-demarcated paracentral visual field defects and characteristic neurodegenerative alterations of the macula are typically observed [11], [12], [13]. The latter may appear as tissue defects apparent on SD-OCT assessment within both the inner and outer neurosensory retina. Investigating a study population of 33 comprehensively phenotyped patients, we observed a general pattern: While structural damage to the outer retina (photoreceptor layers) was accompanied by a loss of retinal sensitivity as detected by microperimetry (Fig. 2, right column; Video S2), similar structural changes limited to the inner retina (proximal to the photoreceptor layer) typically showed preserved function (Fig. 2, middle column; Video S2).

Fixation stability measurements demonstrated that patients with macular telangiectasia type 2 (n = 31) had excellent fixation stability (mean BCEA, 1907 minarc^2^), although having profound paracentral sensitivity loss (see Fig. S1). Consequently, there was no significant difference of fixation stability when compared to a group of normal subjects (n = 15; alpha p = 0.37) corroborating earlier reports [14] and precluding major effects of eye movements on data accuracy.

**Figure 3:**
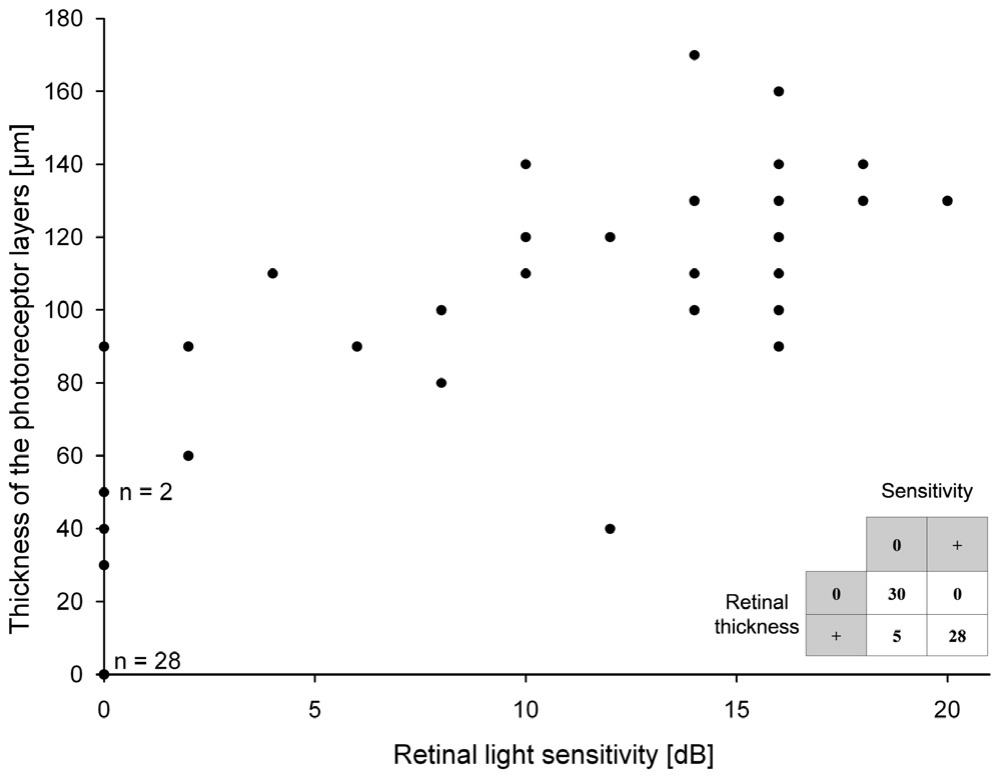
Correlation of retinal light sensitivity (in dB, x-axis) and outer retinal thickness (in µm; y-axis). The cross table compares positive sensitivity and thickness values (i.e. detectable outer retina on SD-OCT images and measurable light sensitivity) with those having a value of zero. There was a significant relationship between the outer retinal thickness and retinal sensitivity (p<0.001; two-sided Fisher exact test of significance). A clustering of data at zero (n = 28) implicates that a complete loss of the outer nuclear layer was associated with an absolute scotoma, i.e. a complete loss of retinal light sensitivity. When omitting this subset in a sub-analysis, outer retinal thickness and retinal sensitivity still showed a significant correlation (r = 0.66; p<0.001; Spearman-Rho; positive scatter plot).

Quantitative analysis revealed that there was a significant relationship between the outer retinal thickness and retinal sensitivity (p<0.001; Fig. 3). The data suggest a biological threshold of about 40–60 µm residual thickness of the parafoveal (1–2 degrees eccentric to the foveal center) photoreceptor layer: Neurodegeneration leaving more than about 50 µm outer retinal thickness would be associated with residual light sensitivity, whereas degeneration beyond this threshold may completely abolish retinal light sensitivity.

### Structure-Function Correlation in Other Retinal Dystrophies

**Figure 4:**
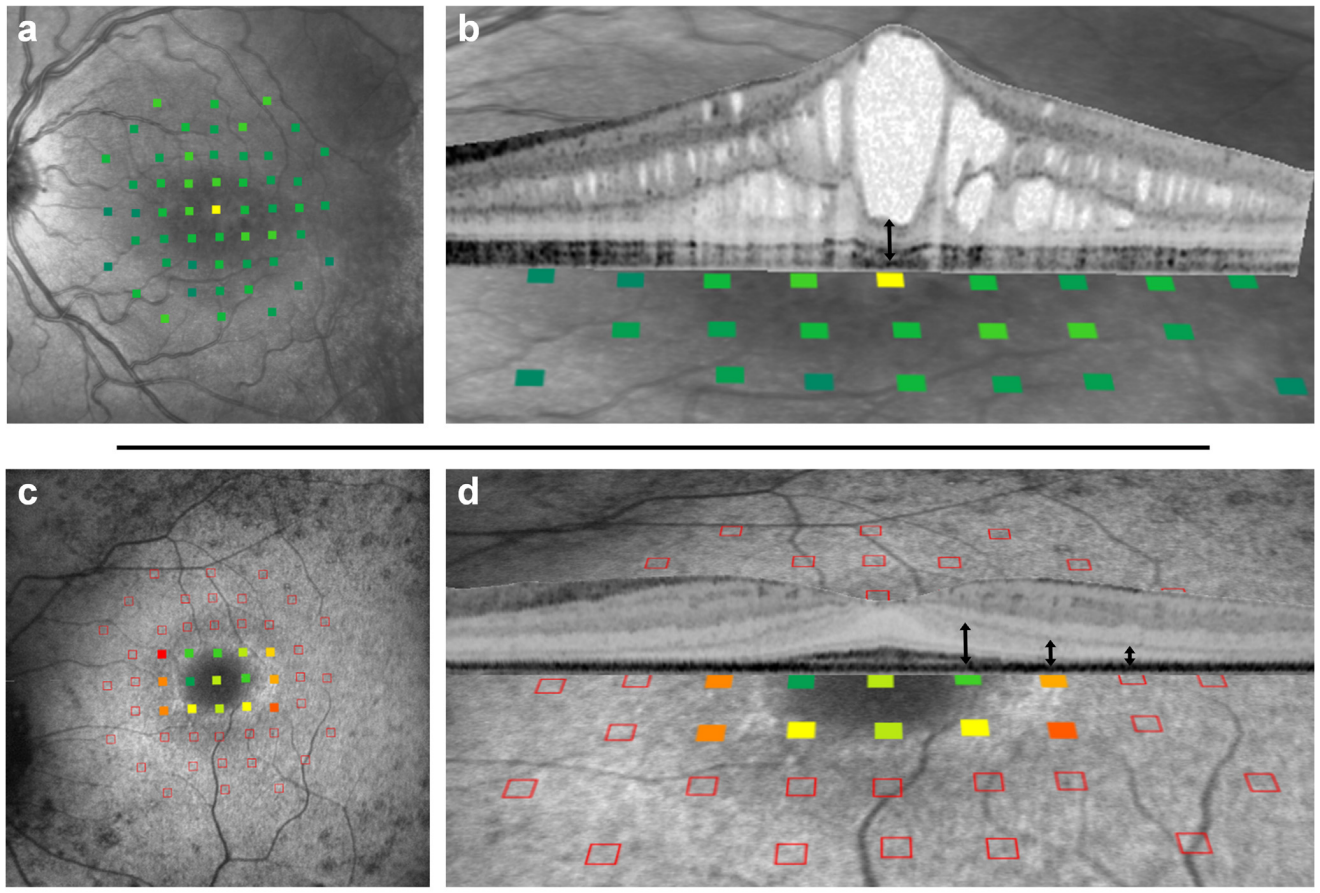
Structure-function correlation of the central retina in a patient with X-linked retinoschisis (upper panels) and a patient with Usher syndrome 2A (lower panels). Retinal sensitivity of the individual testing points is colour coded as in Fig. 2. Upper panels: The functional map derived from microperimetry is superimposed on a cSLO infrared reflectance image (A) and a 15 deg SD-OCT scan (B). Retinal sensitivity is largely preserved despite a splitting of the neurosensory retina mainly confined to layers proximal to the photoreceptors. Only the most distinct alterations result in a relative scotoma (10 dB decreased light intensity compared to the brightest stimulus). The thickness of the corresponding photoreceptor layer (double headed arrow) is about 100 µm. Lower panels: The functional map superimposed on a cSLO fundus autofluorescence image (C) and a cutout of a 30 deg SD-OCT scan (D). Retinal sensitivity is relatively preserved within the parafoveal ring of increased autofluorescence. Peripheral to this ring, the photoreceptor layer reveals a marked thinning. Thickness values at the location of the three perimetric testing points marked with double headed arrows (from left to right) are ∼110 µm, ∼70 µm and ∼50 µm.

The wider applicability of these findings is demonstrated in patients with other neurodegenerative diseases of the retina. Fig. 4 presents two patients suffering from two different monogenic retinal dystrophies. The upper panels (A,B) displays the macula of a 17 year old patient with X-linked retinoschisis (OMIM 312700) carrying a point mutation in exon 4 of the *RS1* gene (c.293C>A). In this disease, it was recently shown that the major pathological alterations of the central retina occur mainly proximal to the photoreceptor layer [15]. In line with these findings, SD-OCT shows extensive inner retinal alterations, leaving an outer retinal thickness of 90–100 µm. Again, there was remarkable functional preservation: Visual acuity was 20/25 and retinal sensitivity was normal except for a relative scotoma at the foveal center. The lower panels of Fig. 4(C,D) illustrates findings in a 33 year old female patient suffering from Usher syndrome 2A (OMIM 608400) carrying a mutation in exon 13 of the *USH2A* gene (c.2299delG). Usher syndrome is the most frequent cause of inherited combined deafness and blindness. The characteristic photoreceptor dystrophy is illustrated on the SD-OCT scan through the fovea: the parafoveal ring of slightly increased autofluorescence delineates a central area of largely preserved retinal function and anatomy from a more eccentric area with marked loss of function and outer retinal thinning. Again, photoreceptor layer thickness of 50 µm or less was associated with an absolute scotoma. The structure-function correlations in these patients with X-linked retinoschisis and Usher syndrome 2A corroborate the findings in macular telangiectasia type 2, namely functional preservation if neurodegeneration spares the photoreceptors, but a strong functional impact if neurodegeneration affects the outer retina.

### Structure-Function Correlation in an Interventional Trial: Ranibizumab to Treat Macular Telangiectasia Type 2

**Figure 5:**
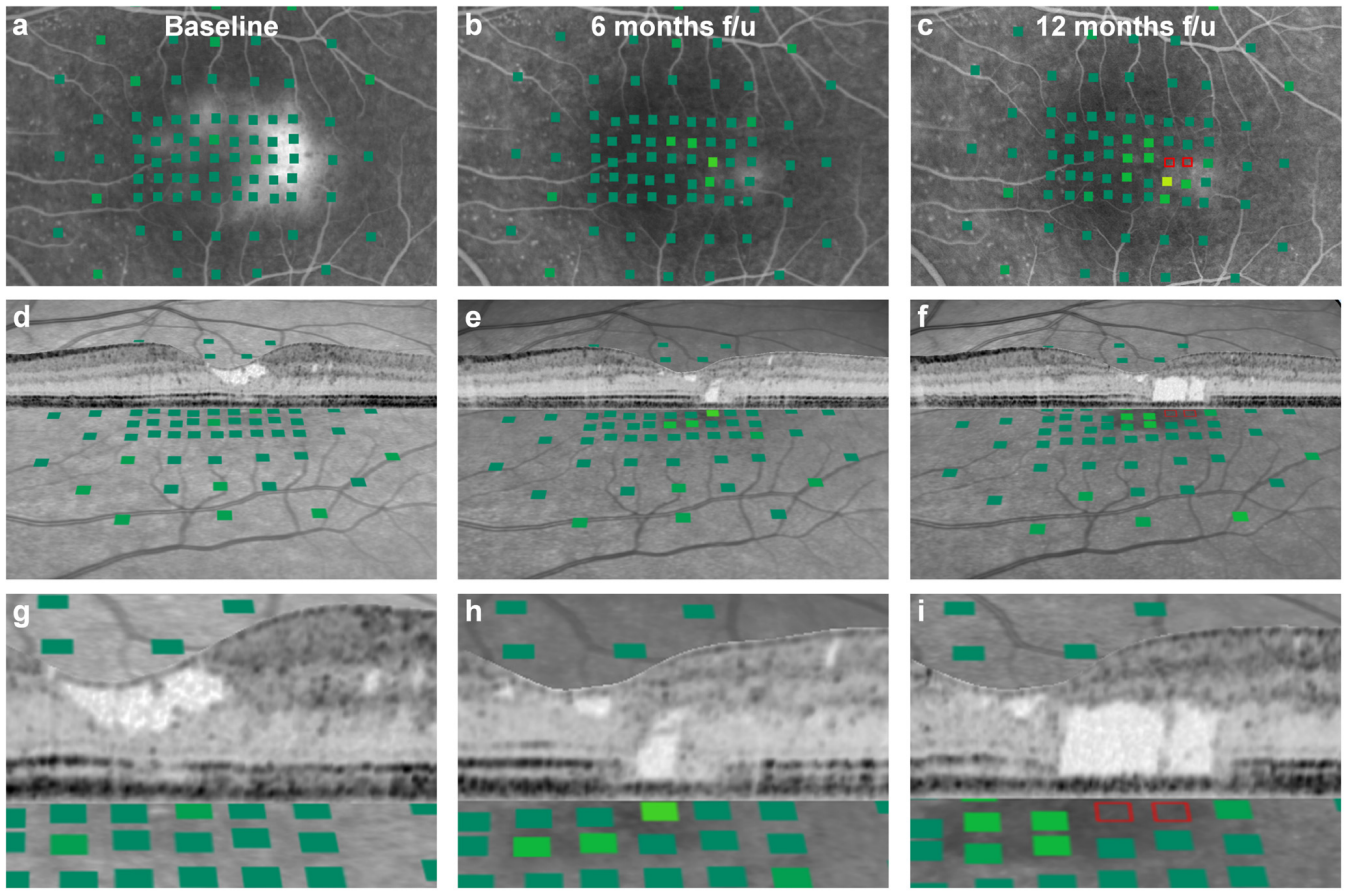
Longitudinal structure-function correlation in macular disease. Retinal sensitivity of the individual testing points is colour coded as in Fig. 2. The 54 year old female patient was treated with 0.5 mg intravitreal ranibizumab in monthly follow-up (f/u) intervals. Shown are images obtained at baseline (left column), after 6 (middle column) and 12 monthly treatments with ranibizumab (right column). The first row shows functional maps (colour coded as in Fig. 1) superimposed on late-phase fluorescein angiography cSLO images. The patient shows leakage of dye in the macula with a maximum at the paracentral temporal area at baseline. Treatment with monthly ranibizumab considerably reduced such leakage (panels b, c). However, the patient developed loss of retinal sensitivity and showed an absolute scotoma (open red rectangles) at the completion of the study period (c). The second and third row (enlarged cut-outs of the middle row) show corresponding SD-OCT images superimposed on back-tilted infrared cSLO images and the same functional map as shown in the upper row. At baseline, the patient shows considerable abnormality of inner retinal anatomy (hyporeflective tissue defect; panels d, g). Retinal sensitivity was normal. After six months of treatment, outer retina abnormalities developed affecting the photoreceptor layer (panels e, h). This alteration measuring about 100 µm appeared to be too small to be detected by fundus-controlled microperimetry. However, after 12 months of treatment, damage of the outer retina was associated with a strong loss of retinal sensitivity (f, i). Visual acuity at baseline was 20/40 and remained unchanged over the study period despite such pronounced loss of paracentral visual function.

Longitudinal observations of the association between morphological abnormalities derived from high-resolution retinal imaging and visual function was investigated in a prospective interventional trial (RAMA trial, see Methods). The study eye of ten patients with macular telangiectasia type 2 received monthly intravitreal injections of 0.5 mg ranibizumab to neutralize VEGF-A over a study period of 12 months, while the other affected eye served as control. Despite a clear effect on retinal morphology (see Fig. S2), there was no significant effect on the primary endpoint, change in visual acuity after 12 months (study eyes: +3.5 letters, SD ±5.4, p = 0.07; fellow eyes: +3.7 letters, SD ±5.9, p = 0.08). In contrast, analysis of the mean change in paracentral retinal sensitivity from baseline to 12 months using microperimetry revealed functional loss in the study eye (−2.3 dB; SD 2.4; p = 0.01) and in the fellow eye (−1.3 dB; SD 1.6; p = 0.03). As exemplified in Fig. 5, high-resolution analysis of the structure-function correlation revealed progressive tissue loss within the photoreceptor layer as the morphological correlate of the decrease in retinal sensitivity. Notably, the example illustrates that minimal outer retinal damage (diameter of about 100 µm) six months after the baseline visit was not detected by microperimetry. After another six months, the enlarged pathology (diameter of about 680 µm) resulted in pronounced paracentral sensitivity loss that left visual acuity unaffected. This pattern of detecting minimal morphologic abnormalities preceding the detection of initial functional defects was consistently observed in the study population.

## Discussion

In previous attempts to correlate retinal function and structure, visual acuity was correlated with central retinal thickness derived from OCT measurements, e.g. in diabetic macular edema. However, the relationship was found to be poor and it was concluded that retinal thickness is a poor surrogate for visual acuity [16]. Using SD-OCT and an automated segmentation technique, it was recently shown that the correlation between visual acuity and specifically the outer retinal thickness is considerably stronger than for full macular thickness suggesting that detailed image analysis is essential to find a correlation [17]. Since visual acuity testing exclusively reveals foveal function but does not provide a functional map of the retina, kinetic or automated static perimetry has been used to reveal and quantify functional defects of the visual field. Using these techniques, there was a consistent relationship between the thickness of the photoreceptor layers as determined by SD-OCT and retinal sensitivity in retinitis pigmentosa, which is genetically heterogeneous group of photoreceptor degeneration [18], [19].

However, these techniques do not provide an accurate correlation between retinal structural pathology and functional defects. Co-registration of microperimetry-, cSLO- and SD-OCT datasets now provides an exact overlay of functional and structural exams and reveals the functional impact of microstructural alterations *in vivo*.

The cross sectional data show that there can be remarkable functional preservation if the pathology spares the outer neurosensory retina that accommodates the process of phototransduction, highlighting the eminent importance of photoreceptor layer integrity for maintaining visual function [18], [19], [20]. Notably, anti-VEGF-A therapy appeared to be ineffective to prevent such functional loss in the longitudinal interventional clinical trial. Actually, recent studies showed that VEGF-inhibition can cause dysfunction and damage of the murine retina [21], [22].

There has long been the fundamental question about whether morphological changes in the neurosensory system precede functional alterations or vice versa. Our data may suggest that high-resolution imaging can detect very early retinal pathology before its progression causes functional loss as detected by functional mapping. It cannot unequivocally be decided whether there are compensatory functional mechanisms when there are sufficiently small morphological defects or whether microperimetry is methodologically limited to detect the earliest functional deficits, or both. In either case, high-resolution OCT appears superior to functional mapping to detect relevant damage of the central retina early in the disease process.

cSLO topographic imaging of the retina provides approximately 15 µm transversal resolution [23]. However, if aberrations are compensated using adaptive optics, the transversal resolution of a cSLO could be improved to less than 3 µm [24]. Zawadzki et al. using spectral/Fourier domain detection and a closed loop adaptive optics system achieved improved lateral resolution (∼ 4 µm) of retinal images, while maintaining the high axial resolution (∼6 µm) of OCT. Their instrument enabled the three-dimensional visualization of different retinal structures, e.g., microscopic blood vessels, and an en face view of the cone photoreceptor mosaic [25]. Simultaneous cSLO and SD-OCT in combination with adaptive optics may have the potential to provide researchers and clinicians with near cellular-resolution information on intraretinal morphology [2].

Although there has been progress correlating single cone photoreceptors to the response of central visual neurons anatomical maps [26] or relating retinal nerve fiber layer distribution with visual fields in glaucoma [27], this is the first report of a high-resolution structure-function correlation of pathology within the central human retina. Such a methodology is capable of revealing an unprecedented pathophysiological context in retinal disease that is highly relevant for therapeutic decision. In diseases such as X-linked retinoschisis where gene therapy has been shown to work in animal models [28], [29], [30], [31], [32], [33], future therapeutic trials should aim to preserve the outer retinal morphology that, if still intact, may provide the patient with relatively good visual function.

A robust correlation between structural abnormalities of the retina and functional deficits may have to be established for every individual disease entity. For enhanced S-cone syndrome due to mutations in the NR2E3 gene, it has recently been shown that both increased and decreased retinal thickness are associated with retinal sensitivity loss [34]. While an increase in retinal thickness was due to a foveal schisis, a decrease was due to neurodegeneration of the retina. This study shows that retinal thickness per se is a poor predictor of retinal function and it underscores the importance to correlate individual retinal layers with retinal function as performed in our study.

Current clinical practice emphasizes the development of techniques to diagnose disease in its early stages, when treatment is most effective and irreversible damage can be prevented or delayed. There is a strong need to define surrogate endpoints which may serve as a substitute for a true clinical endpoint (such as functional loss) but are more efficient [35], [36]. Since our data indicate consistent associations between high-resolution imaging and visual function and since such imaging techniques are becoming more widely available, high-resolution imaging of the retina may have a high potential to serve as surrogate endpoints in future clinical trials.

## Materials and Methods

### Patients

The prospective observational study received ethical approval by the local institutional review board (IRB). Approval for the prospective interventional clinical trial (see below) was obtained from the IRB, the European Medicines Agency (EMEA, London, UK) and the German competent health authority (Paul-Ehrlich Institut, Berlin, Germany). The diagnosis of macular telangiectasia type 2 was based on typical findings on fluorescein angiography and ophthalmoscopy as described by Gass and Blodi [37]. Additionally, all patients showed a macular pigment distribution and alterations on OCT characteristic for the disease [12], [13], [38], [39]. Thirty-three patients (66 eyes) were enrolled in the study. Three eyes were excluded, either due to unavailability of a microperimetric exam (n = 1) or due to the presence of a neovascular membrane (n = 2), leaving 63 eyes for analysis. Individual patients with retinal dystrophies were selected from a comprehensive cross-sectional study of high-resolution retinal imaging in monogenic retinal dystrophies. The principles of the declaration of Helsinki and good clinical practice guidelines were followed. Patients provided written informed consent for study participation.

### Visual acuity testing

Best corrected distance visual acuity was measured by standard Early Treatment Diabetic Retinopathy Study (ETDRS) protocols with a distance chart transilluminated with a chart illuminator (Lighthouse International, New York, NY).

### Microperimetry

Fundus-controlled microperimetry (MP1, Nidek Technologies, Padova, Italy) was performed as described previously [11]. Briefly, a tracking software uses anatomic landmarks with high contrast, such as retinal vessels, to record eye movements during the examination. This information is used to project the test stimuli (Goldman III size, 100 ms projection time, 4-2 staircase test-strategy) in spatial correlation to anatomic landmarks, independent from eye movements. A predefined automatic 16° macula test pattern with a red cross (2° in diameter) as fixation target was used. In patients with macular telangiectasia type 2, additional stimuli were placed within the macular area between the predefined testing points. This resulted in a maximum of 45 testing points (each 1 degree apart) covering an area of 8 degrees horizontally and 4 degrees vertically centered on the foveal centre. Test points revealing an absolute scotoma as well as individual testing points with questionable validity were retested manually by the examiner.

### Spectral domain optical coherence tomography

SD-OCT was performed with the Spectralis HRA+OCT (Heidelberg Engineering, Heidelberg, Germany). A second laser scanning system (confocal scanning laser ophthalmoscope, cSLO) operates simultaneously and allows recordings of topographic confocal images, including modalities such as near-infrared reflectance, fundus autofluorescence or angiography. The position of the SD-OCT scan on the fundus is in exact anatomical correlation to the topographic cSLO image. Stable positioning despite eye movements is ensured by an eye tracking system that continuously recognizes eye movements using landmarks with sharp contrast boundaries on cSLO images. Averaging of multiple single SD-OCT scans at the same position leads to an increased signal to noise ratio in the generated mean images. The SD-OCT system works with an average wavelength of 870 nm and has an axial resolution of 7 µm and a lateral optical resolution of 14 µm [10].

### Co-registration of functional testing and structural imaging

A custom-made software tool (MultiModalMapper) allows for co-registration of the coordinate systems of microperimetry and OCT datasets. Microperimetry data as well as SD-OCT data are fundus-related and can be mapped by registering the fundus images of both devices. Microperimety data (Nidek MP1) consists of 2 files that can be imported into the software tool. One file contains the actual perimetry data as well as mapping information for the second file, the fundus image. OCT data can be exported from the EyeExplorer (Heidelberg Engineering, Heidelberg, Germany) as binary VOL file containing B-scans, cSLO and B-scan segmentation data (optional). The binary file structure can be imported and selected content can be visualized (cSLO for registration; cSLO and B-Scans for 3D view).

The affine transformation model needed to map microperimetry data (source coordinate system) on OCT data (target coordinate system) is calculated from corresponding landmarks set on both fundus images. Landmark selection is done manually since the quality of the perimetry fundus image turned out to be insufficient for automatic approaches. The result of the co-registration process is visualized immediately as overlay image to allow for adjustment by moving or adding additional landmarks. The applied affine transformation model accounts for adjustable radial lens distortion factors for both the source and the target coordinate system [40]. At least 3 landmarks are needed to solve the linear system for the affine transformation.

The transformed microperimetry data can then be visualized as an additional layer within the 3D view of the SD-OCT. Zoom and rotation of the 3D view as well as scrolling through B-scans and moving the SLO image and SD-OCT scan together with the microperimetry layer are supported. The software tool has been developed as WPF application (Microsoft Windows Presentation Foundation) using the Microsoft. NET Framework 3.5 sp1. The OCT 3D view is implemented as WPF 3D model, whereas B-scans, cSLO image and the microperimetry layers are displayed as textured 3D planes.

The software also allows for the alignment of B-scans using retinal pigment epithelium segmentation data which can be loaded from the VOL file as well as extracted automatically using a Matlab (The MathWorks, Inc) script that processes the B-scans. Segmentation borders are mathematically represented as bsplines that can be altered manually in case of segmentation errors.

### Correlation of retinal sensitivity and outer retinal thickness

Correlation between sensitivity assessed by microperimetry and thickness of the outer retina was tested within an area of 3×3 degrees (nine testing points) centred 2 degrees temporal to the foveal centre. This area was selected due to its vulnerability to functional damage in patients with macular telangiectasia type 2 [11]. The outer retinal thickness was measured between the inner border of the retinal pigment epithelial layer and the border between outer nuclear layer and outer plexiform layer. Thus, the measurement reflects all photoreceptor layers (outer nuclear layer, inner and outer segments) on the OCT images. The thickness was always measured in direct topographic correlation to the lowest sensitivity value of the nine testing points within the above defined area. If several testing points qualified with an equally low value, the measurement was performed at the location of most profound neurosensory pathology. Severely disrupted outer retinal morphology in a subset of patients introduces some noise in image analysis as previously discussed [41]. The outer retinal thickness was set as zero in areas where the outer plexiform layer was directly attached to the retinal pigment epithelium layer or when hyporeflective spaces or intraretinal pigment migration extended throughout the entire photoreceptor layer. To analyze the relationship between the outer retinal thickness and retinal sensitivity a two-sided Fisher exact test of significance was performed. This statistical test was chosen because of a clustering of data points at zero. To investigate if the correlation is still significant when this subset of data points is excluded a Spearman's rank correlation coefficient was calculated as a sub-analysis of the correlation of outer retinal thickness and retinal function.

### Recording of three-dimensional SD-OCT volume scans

In patients with macular telangiectasia type 2, three-dimensional volume scans were recorded using the following settings: a scan pattern consisting of 61 equally spaced horizontal B-scans (768 A-scans per 30°, resulting in a lateral digital resolution of 11 µm/pixel) covered an area of 30 (horizontally) ×25 (vertically) degrees centered on the foveola. In addition, as exemplified in the videos, individual patients were examined using a denser SD-OCT scanning pattern. This encompassed 97 equally spaced horizontal scans covering an area of 15 degrees horizontally ×10 degrees vertically, resulting in about 30 µm distance between individual horizontal scans. Here, each B-scan consisted of 768 A-scans per 15°, resulting in a lateral digital resolution of 5 µm/pixel.

### Assessment of fixation stability

Fundus controlled fixation testing with dilated pupils was also performed with the MP1 prior to microperimetry and retinal imaging. A suprathreshold 2° red cross was used as fixation target against a white background with a luminance of 1.27 cd/m2. The room was dark during examination. Fixation stability was assessed over a period of 30 seconds and calculated as bivariate contour ellipse area (BCEA), within which the centre of the target was imaged 68% of the time. The fixation data were further evaluated to determine if there were multiple fixation loci (preferred retinal loci, PRLs) using a kernal density estimator technique described by Crossland et al. [42]. Data were only recorded whilst the eye tracking was active. Thus, no data were recorded whilst blinking or loss of tracking. Due to the possible dependency between the subject's two eyes only right eyes were included in the analysis. A two-tailed unpaired t-test was used to detect a difference in fixations stability between the patients' group and a group of 15 healthy normal controls.

### Prospective interventional clinical trial (RAMA trial)

Based on short-term observations after intravitreal application of the vascular endothelial growth factor (VEGF) inhibitor bevacizumab in patients with non-proliferative macular telangiectasia type 2 [43], a prospective interventional clinical trial was initiated to study the effectiveness and safety of intravitreal VEGF-inhibition using ranibizumab (ClinicalTrials.gov number, NCT00504400; Paul-Ehrlich-Institut/EudraCT-EMEA, CRFB002ADE04; RAMA trial). Ranibizumab is an antigen binding region fragment of a recombinant, humanized monoclonal antibody which was recently approved by the Food and Drug Administration for the treatment of neovascular age-related macular degeneration. One eye of each patient (n = 10) with a non-proliferative disease stage received a monthly intravitreal injection of 0.5 mg ranibizumab for 12 months. The primary endpoint was the change in best-corrected distance visual acuity after one year compared to baseline (measured using ETDRS distance visual acuity charts). Secondary endpoints included the change in retinal sensitivity measured with microperimetry, the change in retinal thickness assessed by SD-OCT and the change in parafoveal leakage assessed by fluorescein angiography. For the analysis of the change in retinal sensitivity, nine testing points covering an area of 3×3 degrees centred 2 degrees temporal to the foveal centre were analyzed. This area was selected due to its vulnerability to functional damage in patients with macular telangiectasia type 2 [11]. Changes in visual acuity and retinal sensitivity between baseline and the one year follow-up were compared using the two-tailed paired t-test. Mean visual acuity showed a transient increase in the study eye. However, after 12 months of treatment there was no significant change of visual acuity compared to baseline or compared to the fellow eye (see Results). There was a decrease of vascular pathology and leakage detectable on fluorescein angiography (as visualized in Fig. 5: cf. panels b and c with a). This was accompanied by a topographically related significant reduction in macular thickness.

## Supporting Information

Figure S1 Fixation stability in macular telangiectasia type 2 in the presence of profound paracentral retinal sensitivity loss. Illustration of fixation stability in the same patient as presented in Fig. 5 at the 12 month follow up examination. Although there is a deep paracentral scotoma (Fig. 5, right column), fixation is very stable. This is a typical finding in patients with macular telangiectasia type 2 as reported recently (4). The red cross with a diameter of 2 degrees of visual angle represents the fixation target. The blue dots visualize fixation at individual time points (shifts in the horizontal, X, and vertical, Y, direction relative to a reference frame) during the examination time of 30 seconds. The spread of the fixation dots centered on the baricenter represents the patient's eye movements. The graph below shows the distances (in degrees) between the fixation points (25 points/sec) and their baricenter vs. time.
(2.43 MB TIF)

Figure S2 Morphological effect of anti-VEGF therapy in macular telangiectasia type 2. Late phase fluorescein angiography in a patient with macular telangiectasia type 2 at baseline (left panel) and one month after the first intravitreal injection of 1.25 mg ranibizumab (middle panel). The right panels show the corresponding SD-OCT analysis. Green colour coding marks an area of retinal thinning. The right lower panel depicts the thickness profile along the white dashed line in the left panel. Anti-VEGF therapy has a clear morphological effect in macular telangiectasia type 2: There is a decrease in late phase angiographic leakage that is topographically related to a decrease in retinal thickness.
(6.19 MB TIF)

Video S1 Structure function correlation in a normal subject.
(9.44 MB MOV)

Video S2 Structure function correlation in a patient with macular telangiectasia.
(8.72 MB MOV)
