# Supplementary figures and images for: Structure-Function Correlation of the Human Central Retina
Source: PLoS One. 2010 Sep 22;5(9):e12864. doi: 10.1371/journal.pone.0012864 (PMC2943911; doi:10.1371/journal.pone.0012864)

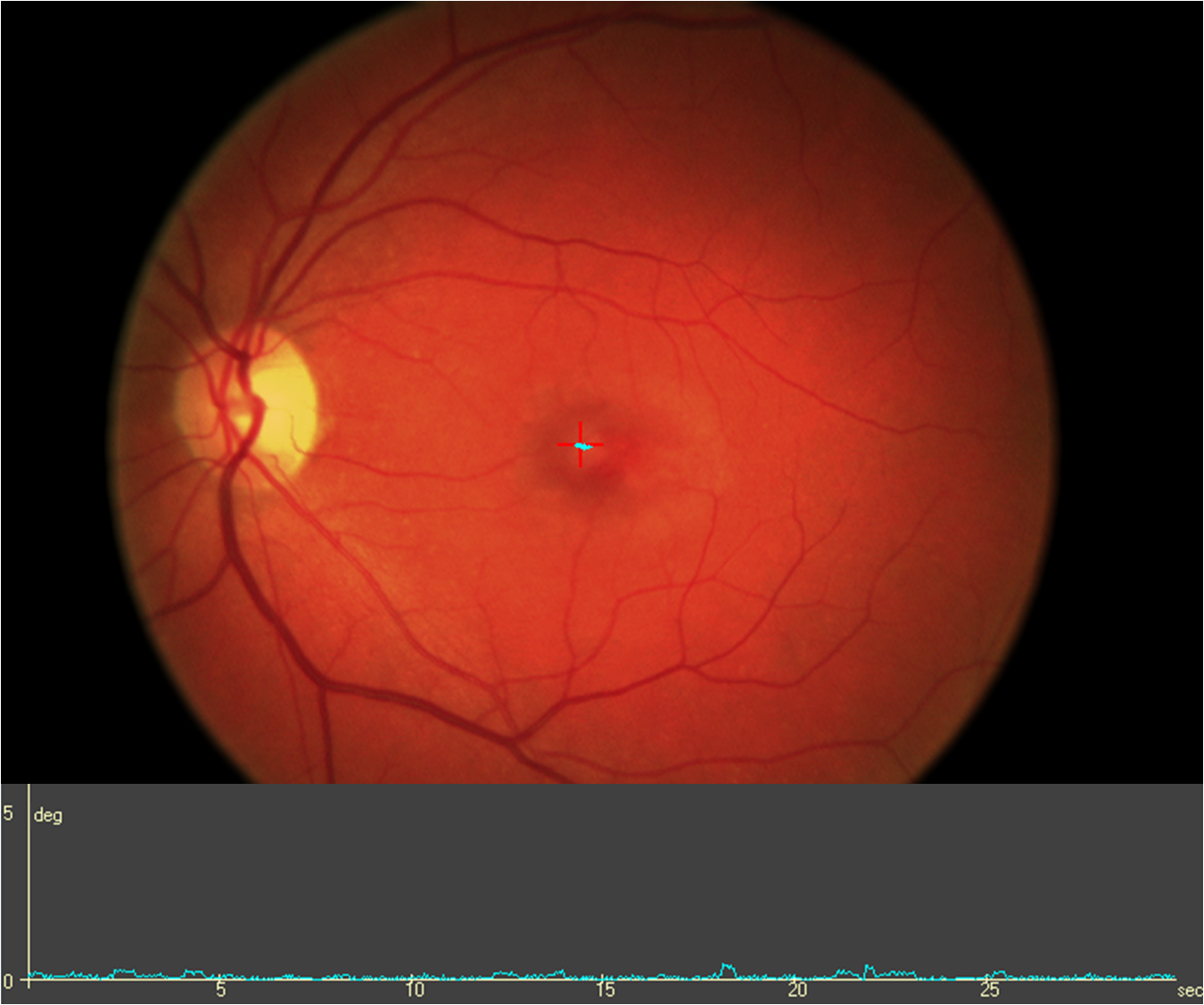

Supplement: Figure S1 — Fixation stability in macular telangiectasia type 2 in the presence of profound paracentral retinal sensitivity loss. Illustration of fixation stability in the same patient as presented in Fig. 5 at the 12 month follow up examination. Although there is a deep paracentral scotoma (Fig. 5, right column), fixation is very stable. This is a typical finding in patients with macular telangiectasia type 2 as reported recently (4). The red cross with a diameter of 2 degrees of visual angle represents the fixation target. The blue dots visualize fixation at individual time points (shifts in the horizontal, X, and vertical, Y, direction relative to a reference frame) during the examination time of 30 seconds. The spread of the fixation dots centered on the baricenter represents the patient's eye movements. The graph below shows the distances (in degrees) between the fixation points (25 points/sec) and their baricenter vs. time. (2.43 MB TIF) [file pone.0012864.s001.tif]

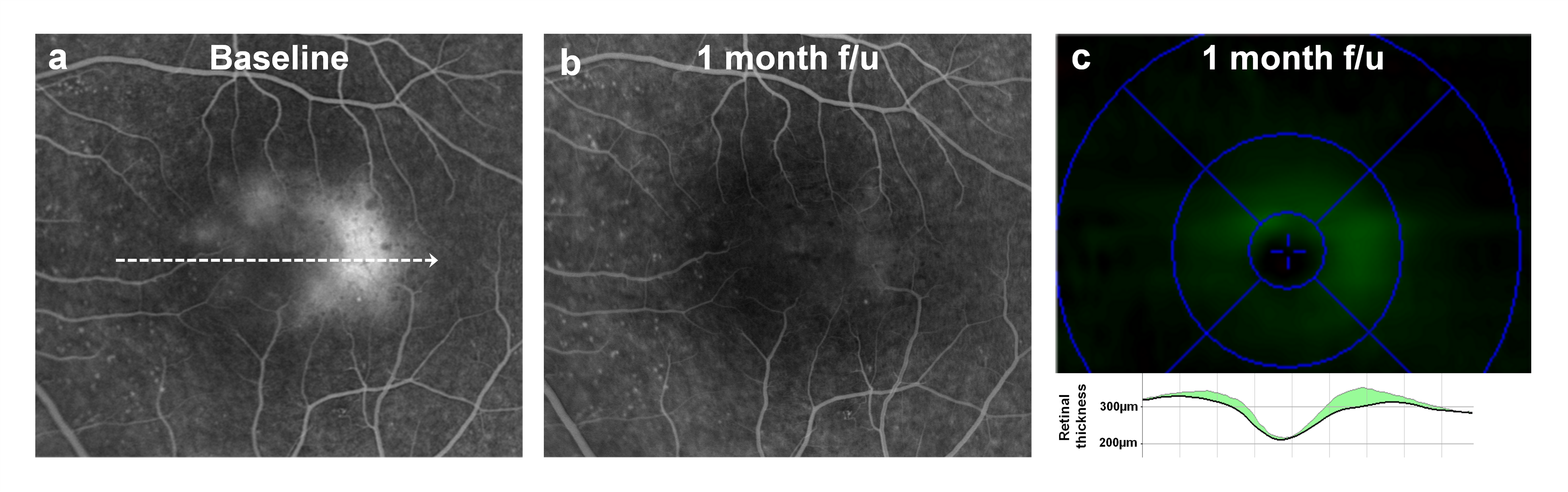

Supplement: Figure S2 — Morphological effect of anti-VEGF therapy in macular telangiectasia type 2. Late phase fluorescein angiography in a patient with macular telangiectasia type 2 at baseline (left panel) and one month after the first intravitreal injection of 1.25 mg ranibizumab (middle panel). The right panels show the corresponding SD-OCT analysis. Green colour coding marks an area of retinal thinning. The right lower panel depicts the thickness profile along the white dashed line in the left panel. Anti-VEGF therapy has a clear morphological effect in macular telangiectasia type 2: There is a decrease in late phase angiographic leakage that is topographically related to a decrease in retinal thickness. (6.19 MB TIF) [file pone.0012864.s002.tif]
